# Understanding the Intricate Pathophysiology of Psoriasis and Related Skin Disorders

**DOI:** 10.3390/ijms26020749

**Published:** 2025-01-17

**Authors:** Olguța Anca Orzan, Cristina Violeta Tutunaru, Simona Laura Ianoși

**Affiliations:** 1 Faculty of Medicine, ‘Carol Davila’ University of Medicine and Pharmacy, 020021 Bucharest, Romania; olguta.orzan@umfcd.ro; 2 Department of Dermatology, ‘Elias’ University Emergency Hospital, 011461 Bucharest, Romania; 3 Department of Dermatology, Faculty of Medicine, University of Medicine and Pharmacy of Craiova, 200349 Craiova, Romania; simonaianosi@hotmail.com

**Keywords:** psoriasis–atopic dermatitis overlapping syndrome, psoriasis and vitiligo concomitant disease, psoriasis and alopecia areata overlap, psoriasis concomitant skin disorders

## Abstract

Psoriasis is a chronic inflammatory condition that is polygenic and multisystemic, impacting approximately 2–3% of the global population. The onset of this disease is influenced by an intricate interplay of genetic and environmental factors, predisposing individuals to the psoriasis phenotype. The complex pathogenesis of psoriasis contains certain key aspects found in other autoinflammatory and autoimmune dermatological diseases. Among these, vitiligo, alopecia areata, hidradenitis suppurativa, vitiligo, connective tissue diseases, bullous dermatoses, and atopic dermatitis are conditions that share overlapping immune system dysfunction, making their relationship with psoriasis particularly significant. For our research, we explored various terms including “shared”, “concomitant”, “coincident”, “overlap”, “coexist”, and “concurrent”, in relation to conditions such as “psoriasis”, “alopecia areata”, “hidradenitis suppurativa”, “atopic dermatitis”, “vitiligo”, “bullous pemphigoid”, “pemphigus vulgaris”, “lupus erythematosus”, “dermatomyositis”, and “systemic sclerosis.” Additionally, we used specific search queries like “atopic dermatitis overlapping syndrome” and “psoriasis and vitiligo concomitant disease” in the PubMed and Web of Science databases. While distinct in their clinical presentation, the skin diseases related to psoriasis may become associated, complicating diagnosis and treatment. In this narrative review, the complex pathophysiology of psoriasis is described, along with its close relationship to other skin conditions. This review provides an exhaustive description of both immunological and non-immunological pathways contributing to their development. Understanding the intricate interconnection between psoriasis and these conditions is of interest to scientists in developing novel research directions and to clinicians in providing holistic care, as managing one condition may influence the course of others.

## 1. Introduction

Psoriasis is a chronic inflammatory condition that is polygenic and multisystemic, impacting approximately 2–3% of the global population. The onset of this disease is influenced by an intricate interplay of genetic and environmental factors, predisposing individuals to the psoriasis phenotype. Psoriasis is associated with other dermatological conditions, including alopecia areata, vitiligo, hidradenitis suppurativa, chronic spontaneous urticaria, bullous dermatoses, and various autoimmune diseases [1].

While psoriasis is prevalent worldwide, its incidence varies considerably among different populations. In the United States, approximately 2% of the populace is affected. Notably higher rates have been documented in regions such as the Faroe Islands, where 2.8% of the population exhibits the disease. Conversely, certain ethnic groups, including individuals of Japanese descent, report low prevalence rates, and the condition may be absent among Aboriginal Australians and Indigenous populations from South America [1].

Psoriasis can manifest at any age; cases have been recorded at birth and in elderly individuals [1]. However, accurately determining the age of onset poses challenges, as many studies depend on the patient’s recollection of symptom onset or the physician’s diagnosis at the initial consultation. This reliance can result in inaccuracies, as some patients may experience minimal symptoms for an extended period before seeking medical attention. The age of onset of psoriasis seems to have a double-peaked distribution. The average age at which the disease first appears typically ranges from 15 to 20 years, with a second peak occurring between the ages of 55 and 60. Two distinct clinical types of psoriasis have been identified: type I and type II. Type I occurs in individuals aged 40 or younger and accounts for more than 75% of cases, while type II manifests in individuals over the age of 40. Patients with early-onset psoriasis generally report a higher familial incidence and tend to experience more severe disease than those with late-onset psoriasis. Furthermore, early-onset psoriasis has been strongly associated with the human leukocyte antigen (HLA)-Cw6 [1].

This review aims to provide a comprehensive understanding of the complex pathophysiology of psoriasis and its associated dermatologic conditions. To support our research, we searched various terms, such as “shared”, “concomitant”, “coincident”, “overlap”, “coexist”, and “concurrent”, in combination with “psoriasis”, “alopecia areata”, “hidradenitis suppurativa”, “atopic dermatitis”, “vitiligo”, “bullous pemphigoid”, “pemphigus vulgaris”, “lupus erythematosus”, “dermatomyositis”, and “systemic sclerosis”. Examples of specific search queries included “psoriasis–atopic dermatitis overlapping syndrome” and “psoriasis and vitiligo concomitant disease” in the PubMed and Web of Science databases, reinforcing the focus of this narrative review.

## 2. Genetic Background of Psoriasis: Key Loci and Advances in Genome-Wide Association Studies (GWAS)

Genetic factors have long been acknowledged as critical components in the etiology of psoriasis. The prevalence of psoriasis is markedly higher among the affected individuals’ first- and second-degree relatives than the general population. Furthermore, the condition exhibits greater concordance among monozygotic twins than dizygotic twins [2].

Linkage studies have identified at least nine genomic regions (loci) associated with psoriasis, called PSORS1 through PSORS9. The PSORS1 region, which is located within the class I interval of the major histocompatibility complex (MHC), primarily encodes genes implicated in antigen presentation. This region also includes the gene corneodesmosin, which encodes a desmosomal protein that is essential for keratinocyte cohesion and desquamation. The PSORS1 locus accounts for approximately 35–50% of the heritability of the disease explained by known loci.

Additionally, the PSORS2 and PSORS4 loci, situated on chromosomes 17q25 and 1q21, respectively, have been associated with the development of psoriasis. The CARD14 gene, located within the PSORS2 locus, encodes an activator of nuclear factor-κB (NF-κB) and harbors variants linked to rare and common psoriasis forms. Proteins involved in the terminal differentiation of the stratum corneum are encoded by genes linked to the late cornified envelope found in the PSORS4 locus. Genome-wide association studies (GWAS) spanning Chinese and European populations have linked this region to psoriasis susceptibility.

GWAS employ highly optimized microarrays that enable efficient genotyping of millions of genetic markers across the genome. Given sufficiently large sample sizes, GWAS facilitates the detection of even subtle differences in allele frequencies between affected individuals and unaffected controls, thereby proving to be a more potent method than linkage analysis. As a result, GWAS has profoundly influenced the genetic investigation of common complex diseases such as psoriasis [2].

However, one intrinsic limitation of GWAS is that it primarily reveals statistical associations. The genetic variants identified may, due to linkage disequilibrium, merely serve as proxies for distinct “causal” variants that exert biological effects and influence disease risk. To enhance GWAS signals and identify potential causal susceptibility alleles, researchers have utilized genotyping arrays with dense coverage in relevant regions.

Moreover, the integration of datasets from international collaborations through meta-analyses of GWAS has proven essential for augmenting statistical power and identifying novel loci associated with disease susceptibility [2].

## 3. Pathogenic Pathways Across Psoriasis Subtypes

Various triggering factors can elicit the onset of psoriasis in genetically predisposed individuals and may also facilitate consequent exacerbations of the disease. These may be grouped into extrinsic and intrinsic factors (Table 1) [3].

Drug-related psoriasis refers to the onset or worsening of psoriasis linked to certain medications. Some drugs can exacerbate existing psoriasis, induce psoriatic lesions on previously unaffected skin in patients who already have the condition, or even trigger the disease in individuals without a family history of psoriasis and those who are predisposed to it. Identifying drug-related causes of psoriasis can be challenging in clinical settings due to significant variability in the latency period between medication initiation and psoriatic skin lesions’ appearance.

Clinically and histopathological, drug-related psoriasis may closely resemble typical psoriasis. Drug-related psoriasis can present in various forms including plaque-type, palmoplantar, nail, scalp, pustular, and erythrodermic psoriasis. In most cases, the histopathological findings in drug-related psoriasis are nearly indistinguishable from those of conventional psoriasis. However, eosinophilic infiltrates in the dermis and lichenoid reactions can sometimes indicate drug-related psoriasis. Unlike non-drug-related psoriasis, in which the capillaries in the upper dermis appear convoluted and tortuous, this characteristic may not be evident in drug-related psoriasis. Additionally, there may be differences in the formation of neutrophilic micro-abscesses in the stratum corneum of the epidermis. While these clues can guide the diagnosis, they are not the most crucial indicators.

Commonly recognized drugs that can trigger the onset or flare-up of psoriasis include β-blockers, lithium, antimalarial drugs, and interferons, as well as angiotensin-converting enzyme inhibitors, among others (Table 2) [3].

The pathways responsible for drug-related psoriasis are highly complex, but the exact mechanisms remain elusive. Certain medications have been shown to affect keratinocyte hyperproliferation and the IL-23/IL-17 signaling pathway. Cyclic adenosine monophosphate (cAMP) acts as an intracellular messenger that stimulates proteins responsible for cellular differentiation and inhibits proliferation; β-blockers can lead to a decrease in intraepidermal cAMP, resulting in keratinocyte hyperproliferation. Imiquimod-induced skin inflammation is a well-accepted model of psoriasis in animal studies. Imiquimod activates toll-like receptors 7/8, which can induce and exacerbate psoriasis, primarily through the IL-23/IL-17 axis.

Recently, immune checkpoint inhibitors and molecular inhibitors have been used in the treatment of malignancies and autoimmune diseases, and these drugs may influence the immune system, potentially leading to the development of psoriasis. Although psoriasis symptoms are rarely worsened during biologic therapy, they can still be triggered by biologics, resulting in what are known as paradoxical reactions. While most reported paradoxical reactions have been associated with tumor necrosis factor (TNF)-α inhibitors, there is a growing incidence of reactions associated with biologics targeting IL-23 and IL-17. These biologics block immune signaling pathways, potentially causing cytokine imbalances. Paradoxical reactions are believed to result from an imbalance in cytokine production, leading to overproduction of interferon-α and altered lymphocyte recruitment and migration. In patients diagnosed with drug-related psoriasis, suspected medications should be discontinued and replaced with alternative treatments [3].

The development of psoriatic inflammation is influenced by both innate and adaptive immune responses, with innate responses playing a more significant role in chronic plaque psoriasis. One accepted mechanism involves the overexpression of antimicrobial peptides in psoriatic skin, which act as triggers and contribute to the ongoing maintenance of the condition. LL-37, β-defensins, and specific proteins are some of the most studied markers of psoriasis. In the early stages, there is an abnormal reduction in LL-37 and various antimicrobial peptides produced by keratinocytes under stress conditions, such as physical injury. LL-37 is released by damaged keratinocytes and forms complexes with other molecules—mostly genetic material from altered cells around the keratinocyte.

LL-37 has been recognized as a participant in the pathogenesis of psoriasis, particularly through its role in activating plasmatic dendritic cells. This activation stimulates the production of type I interferon (IFN), which plays a crucial role in the phenotypic maturation of myeloid dendritic cells and is important for the differentiation and function of T-helper 1 (Th1) and T-helper 17 (Th17) cells. Th17 cells are specialized populations of CD4+ T cells that produce several key cytokines, including IL-17, IL-22, IL-21, and TNF-α [4].

Cytokines such as IL-6, IL-1β, and IL-23 play a crucial role in activating Th17 cells. These cytokines can lead to chronic inflammation and autoimmunity. However, Th17 cells that are activated by transforming growth factor-beta (TGF-β) and IL-6 are generally less pathogenic; they mainly help maintain tissue integrity and provide defense against pathogens. Additionally, the migration of myeloid dendritic cells into lymph nodes is observed, which is associated with an increase in the production of TNF-α, IL-23, and IL-12.

The maintenance phase of psoriatic inflammation is driven by the activation of adaptive immune responses through specific T cell subsets. Keratinocyte proliferation in the epidermis occurs through two pathways: inflammation mediated by TNF-α, IL-17, and IFN-γ, and the action of LL-37 complexed with DNA, leading to increased production of type I IFNs. These mediators further enhance keratinocyte activation, resulting in the production of LL-37, pro-inflammatory cytokines (including TNF-α, IL-1β, and IL-6), chemokines, and antimicrobial peptides, which together perpetuate chronic inflammation [4]. This cascade promotes keratinocyte proliferation and the production of antimicrobial peptides and chemokines that recruit neutrophils, sustaining skin inflammation.

Plaque-type psoriasis is characterized by an inflammatory pathway involving TNF-α, IL-23, and Th17 cells. Multiple types of IL-17 are produced by various cell types, including hematopoietic cells such as CD8+ T cells (Tc17), invariant NKT cells, γδ T cells, and non-T non-B lymphocytes (known as type 3 innate lymphoid cells), as well as neutrophils. Inflammatory responses are regulated by IL-17A-F cytokines. It has been established that the most critical signaling in psoriasis is mediated by a receptor activated by two cytokines, IL-17A and IL-17F, with IL-17A displaying a stronger effect. Additionally, some intracellular kinases are activated, leading to the expression of pro-inflammatory cytokines, chemokines, and antimicrobial peptides. Th1 and Th2 cytokines operate through Janus kinase (JAK)-STAT signaling pathways, whereas Th17 responses are mediated by ACT1 and NFκB. Interestingly, γδ T cells can produce IL-17A independently of the IL-23 stimulus [4,5].

The second most common type of psoriasis is pustular psoriasis, which is marked by multiple coalescing sterile pustules. In plaque psoriasis, the adaptive immune system plays a greater role in pathogenesis, with promising results from therapies targeting these elements. In contrast, the innate immune system appears to play a more significant role in pustular psoriasis, making therapies used for plaque psoriasis less effective. There are significant differences in the general pathogenesis of these types of psoriasis, despite some shared metabolic pathways. Generalized pustular psoriasis (GPP) presents with an acute and rapidly progressive course, characterized by widespread redness and subcorneal pustules, often accompanied by systemic symptoms. GPP seems to primarily depend on the activities of keratinocytes, neutrophils, and monocytes, with increased expression of IL-1β, IL-36α, and IL-36γ observed in pustular psoriasis compared to plaque psoriasis. This overexpression of IL-36 appears to be a central mechanism promoting neutrophil accumulation in the epidermis. The significant presence of neutrophil chemokines CXCL1, CXCL2, and CXCL8 (IL-8) aligns with the proposed pathogenesis of GPP [4].

Another less common type is guttate psoriasis, characterized by small erythematous plaques and more commonly seen in children and teenagers. There is currently no evidence demonstrating any differences in the pathophysiologic mechanisms compared to plaque-type psoriasis. It has been suggested that streptococcal superantigens may stimulate the proliferation of T cells in the skin in individuals with guttate psoriasis. Notably, some homology exists between streptococcal proteins and keratin proteins involved in pathways regulated by IL-17. Molecular mimicry may also play a significant role in patients with the major histocompatibility allele HLA-Cw6, as CD8+ T cell IFN-γ responses can be triggered by K17 and M6 peptides in these patients.

Inverse psoriasis is a less common type characterized by affecting intertriginous areas and is generally more erosive than the erythematous plaques seen in plaque psoriasis. Currently, no existing data shows any differences in the pathophysiologic mechanisms between common psoriasis and inverse psoriasis. However, it is speculated that there is a decrease in the number of specific immune cells in the plaques of inverse psoriasis, potentially due to the constant microenvironmental influences.

Erythrodermic psoriasis is an acute condition in which the entire body surface is erythematous and inflamed and is accompanied by serious hemodynamic and metabolic disturbances. However, there is no data to reveal differences between the pathophysiologic mechanism of erythrodermic psoriasis versus common psoriasis [4].

## 4. Psoriasis and Associated Dermatologic Disorders

The intricate and complex pathogenesis of psoriasis contains certain key aspects also found in other autoinflammatory and autoimmune dermatological diseases. Among these, vitiligo, alopecia areata, hidradenitis suppurativa, connective tissue diseases, bullous dermatoses, and atopic dermatitis are conditions that share overlapping immune system dysfunction, making their relationship with psoriasis particularly significant. These diseases, while distinct in their clinical presentation, may become associated in some patients, complicating both diagnosis and treatment. Understanding the intricate interconnection between psoriasis and these conditions is essential for clinicians to provide holistic care, as managing one condition may influence the course of others.

Alopecia areata (AA) is a chronic, inflammatory, immune-mediated condition marked by non-scarring hair loss in distinct, well-defined patches. The disorder impacts individuals of all races and genders equally, though data suggest non-Caucasian individuals may be more susceptible, and men are often diagnosed earlier than women. AA can occur at any age, but 70–80% of patients show symptoms before age 40, with the highest prevalence (10–50%) observed in children, especially those with a family history of the condition [6].

Psoriasis and AA are complex autoimmune conditions shaped by genetic, environmental, and immune factors. Both diseases involve similar immune dysfunction mechanisms. IL-17 and IL-23 are critical cytokines to their pathogenesis. IL-17, primarily produced by CD4+ Th17 cells and certain innate lymphoid cells, is a key proinflammatory cytokine implicated in various inflammatory diseases, including psoriasis, atopic dermatitis, hidradenitis suppurativa, alopecia areata, pityriasis rubra pilaris, pemphigus, and systemic sclerosis. Psoriasis involves chronic inflammation driven by IL-17-producing Th17 cells. Similarly, in AA, IL-17 contributes to inflammation and hair follicle loss, with elevated levels observed in skin lesions and blood. IL-17A contributes by promoting inflammatory responses in both conditions, driving immune cell activation and tissue inflammation. IL-23 enhances IL-17 production through the activation of Th17 cells. The shared pathways involving the IL-23/IL-17 axis highlight potential targets for therapeutic interventions in both diseases. Another key player in psoriasis and AA is represented by dysfunctional Tregs. In the first condition, they exacerbate inflammation and promote IL-17 production. In the second one, CD8+ T cells attack hair follicles, with reduced Treg function leading to inadequate control of this autoimmune response. Both conditions show significant T cell and Treg dysfunction, suggesting that therapies targeting Tregs might benefit both diseases [7].

In psoriasis, IL-17 levels are elevated in both lesional and nonlesional skin, produced by Th17 cells, neutrophils, mast cells, and others. This cytokine, along with IL-22 and TNF-α, creates a proinflammatory milieu that drives keratinocyte proliferation and skin inflammation through NF-κB and MAPK pathways. In AA, there is an infiltration of CD8+ T cells and increased levels of cytokines such as IFN-γ, TNF-α, IL-17, and IL-4. Elevated serum levels of IL-2, IFN-γ, IL-10, IL-13, and IL-17A are observed, with a notable decrease in transforming growth factor-β1. While both psoriasis and AA exhibit increased Th17 cell frequencies and elevated IL-17 levels, AA has shown limited response to IL-17-targeted therapies, indicating a divergent response to cytokine-targeted treatments. The shared involvement of IL-17, IL-22, and TNF-α in psoriasis and AA underscores a common inflammatory pathway, though the therapeutic responses vary [8].

Granzyme B, a serine protease, plays a significant role in autoimmune skin disorders such as psoriasis and AA. In psoriasis, granzyme B contributes to the inflammatory processes and the proliferation of skin cells, exacerbating the disease’s characteristic symptoms such as scaling and redness. In AA, it is involved in the destruction of hair follicles, resulting in the characteristic patchy hair loss. Both conditions share features of chronic inflammation and immune dysregulation, with granzyme B mediating cellular apoptosis and inflammation in each. In psoriasis, granzyme B drives keratinocyte hyperproliferation and inflammation, whereas in AA, it facilitates autoimmune-mediated follicular destruction. Understanding the role of granzyme B in these conditions may provide insights into common pathogenic mechanisms and potential therapeutic targets for managing these chronic autoimmune skin diseases [9].

Autophagy, a cellular process essential for degrading and recycling damaged organelles and proteins, is important for maintaining skin health and managing various skin diseases, including psoriasis and AA. This process helps preserve the skin barrier by regulating cellular turnover and responding to stress. In psoriasis, impaired autophagy can worsen the disease by failing to control cellular damage and inflammation effectively. This leads to increased keratinocyte proliferation and chronic inflammation, contributing to the development and persistence of psoriatic plaques. In AA, autophagy plays a key role in maintaining hair follicle health. Disruptions in autophagy can affect follicle function and immune responses, potentially worsening hair loss and disease progression. Effective autophagy is necessary for managing cellular stress and preserving follicle integrity, with its dysfunction potentially contributing to the onset and severity of AA [10].

The recent identification of coexisting psoriasis and hidradenitis suppurativa (HS) highlights a potential connection warranting further investigation. While numerous case reports describe the simultaneous occurrence of HS and psoriasis, a definitive causal relationship remains unproven due to the lack of robust epidemiological data. Despite their distinct skin manifestations, both chronic conditions share common risk factors, such as obesity and cigarette smoking, and significantly impact quality of life. Additionally, they involve overlapping inflammatory pathways, including the IL-12/IL-23 axis, IL-17 interactions, and TNF-α [11].

HS is a chronic autoinflammatory skin disease characterized by the inflammation of hair follicles, particularly in areas rich in apocrine glands. Its prevalence varies, estimated to be between 0.7% and 1.2%, with a higher incidence in females. HS typically affects intertriginous regions such as the axillary, submammary, and inguinal areas, as well as the anogenital region. This condition can subsequently lead to the formation of abscesses, sinus tracts, and scarring, profoundly impacting the patient’s quality of life.

Psoriasis is thought to be triggered in genetically susceptible individuals following a loss of T cell tolerance to specific self-antigens, potentially in response to an environmental insult that activates innate immune cells. For instance, the release of DNA from damaged cells, when complexed with the antimicrobial peptide LL-37, can activate plasmacytoid dendritic cells (pDCs) via TLR-9, leading to the production of IFN-α. This process enables dendritic cells to present LL-37 autoantigens to T cells. These T cells subsequently differentiate into Th1 and Th17 subsets under the influence of specific polarizing cytokines. Th17 cells produce IL-17A, IL-17F, and IL-22, which, alone or in combination with TNF-α, strongly activate keratinocytes. The activated keratinocytes then secrete antimicrobial peptides, cytokines, and chemokines, which recruit and activate additional immune cells, resulting in a feedback loop of persistent inflammation. The key cytokine, IL-23, is the main mediator in activating and maintaining the inflammatory cascade brought about by Th17 and Th22 cells [11].

On the other hand, the pathogenesis of HS remains incompletely understood, although immune dysregulation is thought to play a crucial role in its etiology. A key event in the development of HS lesions is the occlusion of hair follicles, triggered by hyperkeratosis and hyperplasia of the follicular epithelium, which ultimately leads to cyst formation. Recent studies have identified elevated levels of the cytokines IL-1, IL-23, and IL-17 in HS lesions, suggesting a significant role for Th17 cells in disease pathogenesis.

The initial trigger in the pathogenesis involves the recognition of bacteria and cellular debris as pathogen- and damage-associated molecular patterns (PAMPs and DAMPs) by Toll-like receptors (TLRs) or NOD-like receptors (NLRs) in the dermis, leading to the activation of innate immunity [12]. PAMPs and DAMPs stimulate the inflammasome response, activating the NOD-like receptor protein 3 (NLRP3) in macrophages and neutrophils, which, in turn, leads to the production of caspase-1 and proinflammatory cytokines such as TNF-α and IL-1β. IL-1β induces the release of chemokines, from fibroblasts, which primarily attract neutrophils. TNF-α, produced by macrophages and dendritic cells, upregulates TLRs and stimulates keratinocytes to release various chemokines, including CXCL8, CXCL11, and CCL2, which recruit lymphocytes, neutrophils, and monocytes into the dermis. Additionally, IL-23 and IL-12, produced by activated dendritic cells, promote the polarization of CD4+ T lymphocytes into Th17 and Th1 cells. IL-17, produced by Th17 cells, further enhances macrophage production of IL-1β and TNF-α, amplifying the immune response [12].

In their case series, Chi-Feng Yen and colleagues documented a 3.8% prevalence of psoriasis in patients with HS. All of their patients presented with psoriasis before the onset of HS, suggesting that the flares of psoriasis with hyperkeratosis could induce follicular occlusion, which is considered the primary event in HS [13].

The link between psoriasis and HS is influenced by genetic factors. Both conditions are categorized as autoinflammatory keratinization disorders, characterized by excessive activation of the innate immune system. This activation is influenced by genetic predispositions, including variations in genes such as IL-36RN and CARD14, as well as components of the IL-1 pathway, like IL1RN, TNF receptor 1, and TNFAIP3. The rare genetic disorder known as PsAPASH syndrome, encompassing “Psoriatic Arthritis, Pyoderma Gangrenosum, Acne, and Suppurative Hidradenitis” is believed to stem from mutations in the PSTPIP1 gene. This provides additional support for the theory that genetic factors may underline the association between psoriasis and HS.

The disruption of the skin microbiome, especially the growth of certain bacterial species, such as *Staphylococcus aureus*, may play a crucial role in both HS and psoriasis. Remarkably, the risk of developing HS in patients with more severe or well-established psoriasis may be greater. A hazard ratio (HR) of 2.867 was observed in patients with more than two inpatient visits for psoriasis. Additionally, psoriasis patients with more than two visit records who received a topical corticosteroid prescription had a roughly two-fold increased risk of developing HS. While topical corticosteroids are used to treat psoriasis, prolonged use may damage the follicular structure, which partially explains the predisposition to developing HS. It is a paradox that a 5.914 HR was noted for psoriasis patients with more than two visit records who received a vitamin D prescription. Vitamin D reported benefits for both psoriasis and HS. It was concluded that the prescription of vitamin D analogs indicates a more severe psoriasis, which, in turn, elevates the risk of HS [14].

Due to shared pathogenic mechanisms, biological therapies like adalimumab are effective for both HS and psoriasis. Adalimumab is a monoclonal antibody that binds to TNF and inhibits the interaction of this cytokine with TNF receptors, thereby interrupting the inflammatory cascade. The dosing regimen of adalimumab indicated for treating psoriasis is half of the recommended dosing regimen for HS [4].

When psoriasis occurs in association with HS, the therapeutic strategy should be chosen in concordance with the disease severity bearing in mind that almost all the biologics approved for the treatment of psoriasis are still off-label for HS due to the absence of randomized controlled trials [12].

Both vitiligo and psoriasis share common underlying mechanisms related to immune system dysfunction. While psoriasis involves the rapid turnover of skin cells leading to thick, scaly patches, vitiligo is characterized by the progressive loss of pigment in certain areas of the skin. Despite their distinct clinical presentations, individuals with psoriasis may have an increased risk of developing vitiligo, and vice versa, suggesting a potential link between their genetic factors and immune system pathways. This connection underscores the complexity of autoimmune skin disorders and the need for careful management when both conditions coexist.

In January 2018, Yen, H. and Chi, CC. conducted a search of the MEDLINE and EMBASE electronic databases examining the association between psoriasis and vitiligo. There were 2453 citations identified from the literature search, with a total of 120,866 psoriasis cases and 79,907 vitiligo cases included in this study. There were found significantly increased odds for vitiligo in psoriasis patients (summary OR 2.29, 95% CI 1.56–3.37, studies  =  7), as well as significantly elevated odds for psoriasis in vitiligo patients (summary OR 3.43, 95% CI 1.86–6.33, studies  =  4). The meta-analysis clearly showed that psoriasis and vitiligo are two intertwined conditions associated with each other [15].

In 2017, Sharquie KE, Salman HA, and Yaseen AK conducted a study that aimed to assess the occurrence of vitiligo in psoriasis patients and vice versa. Their research included 1000 participants, comprising 250 individuals with vitiligo, 250 with psoriasis, and 500 healthy controls. Results showed that the frequency of vitiligo among psoriasis patients was 5 (2%), while among healthy controls, the frequency was 3 (0.6%); the frequency of psoriasis among vitiligo patients was 15 (6%), while among healthy subjects, the frequency was 2 (0.4%). Therefore, a statistically significant difference (*p* = 0.001) was observed regarding the occurrence of psoriasis among vitiligo patients. The adjacent relationship between vitiligo and psoriasis was further confirmed by the family history of vitiligo in psoriasis patients and by the family history of psoriasis in vitiligo patients [16].

Chronic spontaneous urticaria (CSU) is defined as the appearance of wheals and/or angioedema for six weeks or more. Referred to as both “chronic urticaria” and “chronic idiopathic urticaria”, this debilitating disorder can have a considerable negative effect on a patient’s quality of life.

Psychological stress significantly impacts skin diseases like psoriasis, atopic dermatitis, acne, and urticaria. Stress can worsen these conditions by disrupting immune balance, impairing skin barrier function, and promoting inflammation. The skin’s complex immune and neuroendocrine systems are involved in this process. In CSU, stress likely worsens symptoms through complex interactions involving mast cells, neuropeptides, and inflammatory cytokines, with hormonal factors like estrogen and progesterone potentially playing a role. Effective management of stress through psychiatric treatments and stress-reducing strategies is crucial for improving symptoms, highlighting the need for an interdisciplinary approach to treating stress-related skin disorders [17].

Research shows that serum levels of IL-31, a cytokine associated with itch, are elevated in patients with CSU and psoriasis compared to healthy controls. Elevated IL-31 levels were specifically observed in psoriasis patients with pruritus, though no clear link was found between IL-31 levels and the severity of the disease or the intensity of the itch. CSU patients with higher antinuclear antibody titters exhibited significantly higher IL-31 levels, suggesting a potential link between IL-31 and autoimmune aspects of CSU. The exact role of IL-31 in these diseases remains unclear, indicating a need for further research [18].

In the systematic review by Widhiati et al. from 2021, several key associations and common mechanisms between psoriasis and urticaria are discussed. Both conditions are marked by chronic inflammation; psoriasis features persistent inflammation with well-defined plaques, while urticaria involves transient wheals and angioedema. The immune system plays a crucial role in both disorders. Psoriasis is driven by T-helper 17 (Th17) cells, whereas urticaria is associated with mast cell activation and immunoglobulin E (IgE) -mediated hypersensitivity reactions. Emerging evidence suggests that disruptions in the gut microbiome can influence systemic inflammation and immune responses, impacting both conditions. Alterations in gut microbiota have been linked to changes in immune function and inflammation in these skin disorders [19].

Mast cells (MCs) play a significant role in the pathogenesis of both psoriasis and CSU. In psoriasis, the role of MCs remains an area of ongoing research. Genetic predispositions and immune responses, involving Th1 and Th17 cells, dendritic cells, NK T cells, macrophages, and keratinocytes, contribute to the disease. In CSU, particularly when complicated by Kounis syndrome, MCs can significantly impact disease progression. Kounis syndrome, characterized by severe allergic reactions or anaphylaxis leading to coronary artery spasms or myocardial infarction due to MCs degranulation, involves various triggers such as certain drugs and foods. Management includes revascularization, corticosteroids, antihistamines, and careful use of vasodilators. Understanding MCs’ roles could offer new therapeutic avenues for managing these complex skin diseases [20]. Both conditions are characterized by neurogenic inflammation, where sensory nerves in the skin release neuropeptides that exacerbate inflammation. MCs play a central role in the pathology of both diseases. In psoriasis, they contribute to chronic inflammation by secreting mediators that promote disease progression. In CSU, MCs are crucial for developing hives through the release of histamine and other inflammatory substances. The interplay between mast cells and sensory nerves is a key mechanism in the chronic inflammation and itch associated with these skin disorders [21].

The analysis of the link between psoriasis and CSU in patients treated with the IL-17A blocker ixekizumab indicates that while this treatment is effective for psoriasis, it can cause urticaria in about 8.8% of patients. In cases of ixekizumab-induced urticaria, IL-17A+ mast cells were found in the skin, which correlated with early relapses of psoriasis after discontinuing the drug. Standard treatments for urticaria, such as antihistamines, did not prevent the relapse of psoriasis, highlighting the need for mast cell stabilizers like cromolyn disodium salt, which have shown promise in reducing inflammation and delaying recurrence in experimental models. Further research is needed to clarify the role of MCs in both conditions and to improve strategies for managing drug-induced urticaria and psoriasis relapse [22].

A paper by Rajagopalan et al. from 2022 examines the role of cyclosporine A in treating psoriasis and CSU, highlighting their shared immunological mechanisms. Both conditions involve immune dysregulation: psoriasis is driven by an overactive Th1/Th17 response leading to chronic inflammation and skin cell proliferation, while CSU is marked by MCs activation and histamine release. cyclosporin A, an immunosuppressive agent, modulates the immune system by inhibiting T-cell activation and cytokine production. This helps reduce inflammation and manage symptoms in both conditions, underscoring cyclosporin A’s effectiveness in addressing the common immune pathways underlying psoriasis and urticaria [23].

Two case studies illustrate how treatment strategies can influence the management of both psoriasis and CSU, highlighting the complex interplay between these conditions and their treatments:

A 36-year-old female with chronic plaque psoriasis and CSU experienced notable psoriasis improvement during CSU flare-ups. Despite moderate plaque psoriasis being only partially managed by various treatments, her psoriasis nearly cleared during CSU exacerbation. This report suggests a possible link between CSU flare-ups and psoriasis remission, though causality remains unclear. MCs activation is a common factor in both conditions and plays a significant role in inflammation. In psoriasis, MCs are generally proinflammatory but can also release anti-inflammatory cytokines and degrade proinflammatory cytokines, potentially influencing psoriasis severity. Previous studies suggest mechanisms such as Hedgehog signaling and neurofibromatosis type 1 gene expression are involved in psoriasis, and antihistamines like ketotifen might suppress psoriatic hyperproliferation by inhibiting MCs degranulation. This case highlights a potential inverse relationship between psoriasis and CSU but underscores the need for further research to clarify this association [24].

A 58-year-old woman with a 30-year history of psoriasis vulgaris and mild bronchial asthma had an inadequate response to methotrexate and acitretin. She developed urticaria while on acitretin, which was partially managed with antihistamines. After seven years, she started treatment with omalizumab for her CSU, followed by secukinumab for psoriasis. This combination led to the remission of CSU and near complete clearance of psoriasis lesions without side effects. Omalizumab (OMZ) is an anti-IgE antibody used for chronic asthma and CSU unresponsive to high doses of H1-antihistamines. It also has anti-inflammatory effects, including normalizing IL-31 levels, which are involved in skin inflammation. OMZ may benefit psoriasis patients, particularly when used in combination with biologics, due to its tolerability and symptom management properties. The connection between psoriasis and urticaria is not well understood but may involve elevated IL-23 levels. Further research is needed to establish the safety and efficacy of OMZ for psoriasis, especially in patients with pruritic symptoms [25].

Autoimmune blistering diseases (AIBDs) are a varied group of disorders caused by circulating autoantibodies that target antigens in the skin and mucous membranes. Bullous pemphigoid (BP) is the most common type of subepidermal autoimmune blistering disease and primarily affects older adults [26]. It is defined by autoantibodies directed against two hemidesmosomal proteins: the transmembrane BP antigen 180 (BP180, also known as collagen XVII) and the intracellular BP antigen 230 (BP230). Similar to psoriasis, BP can be precipitated by various physical factors, such as thermal burns, radiotherapy, and ultraviolet light, as well as chemical factors and a range of pharmacological agents. These include both topical and systemic anti-psoriatic treatments, as well as phototherapy [26].

The link between psoriasis and BP is significant, as studies show that individuals with psoriasis have a 3.05-fold higher risk of developing BP compared to those without psoriasis. Furthermore, over one-third of BP cases in patients with both conditions are diagnosed within one year of the psoriasis diagnosis. Conversely, the incidence of psoriasis in BP patients is 2.5 times higher than in individuals without BP. An Israeli study identified male gender, smoking, and hypertension as risk factors for the concurrent presentation of BP and psoriasis, compared to BP alone. Patients with both conditions tend to exhibit a less severe erosive phenotype, lower levels of pathogenic autoantibodies, and generally present at a younger age [27].

The pathomechanisms underlying the association between psoriasis and BP are likely mediated by autoimmune processes; however, their exact nature remains unclear. The involvement of Th17 cells and the production of IL-17A/F cytokines is a plausible pathogenic link between the two diseases [26].

The pathogenesis of psoriasis vulgaris is primarily driven by proinflammatory cytokines such as TNF-α, IL-23, and IL-17. This is characterized by the upregulation of Th1 and Th17 cell subsets and dysfunction in regulatory T cells. Both innate and adaptive immune responses contribute to the inflammatory process in psoriasis. In the initial stages, antimicrobial peptides and antigenic stimuli activate plasmacytoid dendritic cells, leading to the production of IFN -α, which subsequently activates myeloid dendritic cells. During the maintenance phase of the disease, the TNF-α/IL-23/IL-17 axis is crucial in sustaining the inflammatory response. In particular, IL-23 is essential for inducing the production of IL-17, thereby perpetuating the inflammatory loop [26].

BP is primarily mediated by Th1 and Th2 cells, with a predominance of Th2 responses and the production of autoantibodies against BP180 and BP230 antigens. The binding of these autoantibodies to their target antigens triggers complement activation, mast cell degranulation, and the infiltration of polymorphonuclear leukocytes. This immune response leads to the release of enzymes such as proteases and elastases, which contribute to the separation of the dermal-epidermal junction. Recent research has highlighted the role of Th17 cells in exacerbating the inflammatory response in BP. Additionally, dysregulated regulatory T cells (Tregs) facilitate the activation of autoreactive T cells and the subsequent production of autoantibodies, further contributing to disease pathogenesis. IL-17 and IL-23 were discovered to enhance the expression of proteases responsible for blister formation and the cleavage of the extracellular domain of BP180. Elevated serum levels of these cytokines may serve as prognostic markers, aiding in the identification of BP patients at risk for future relapse. Additionally, IL-17 and IL-23 are crucial for inducing the expression of IL-1β in macrophages from BP patients, with IL-1β subsequently driving inflammasome activation [26,27]. The importance of IL-17A in BP pathogenesis is further supported by findings such as the upregulation of cytokines and related genes in the skin of BP patients, the role of IL-17A in neutrophil activation, and the induction of BP in animal models. Furthermore, IL-23 contributes to BP pathology by promoting the formation of neutrophil-derived DNA extracellular traps, which are known to play a role in the breakdown of immune tolerance in various autoimmune diseases [28].

Although BP is typically characterized by an eosinophilic infiltrate, cases featuring a neutrophil-rich infiltrate have been infrequently reported. According to a retrospective review, 16% of patients showed a significant presence of neutrophils within inflammatory infiltrates. Neutrophilic predominance is particularly noted in cases of drug-induced BP, especially those associated with immune checkpoint inhibitor therapy. Additionally, a recent case linked neutrophil-rich BP with psoriasis. The authors suggested that IL-17A may contribute to the development of neutrophil-predominant blisters [26].

Among the therapies for psoriasis, ustekinumab has been frequently associated with biologic-induced BP, particularly in patients who have had unsuccessful anti-TNF-α treatment. Distinct patterns in the cytokine pathways and clinical course exist between the BP induced by TNF-α blockers and ustekinumab [29].

About one-third of the cases of anti-laminin gamma1 or p200-pemphigoid are associated with psoriasis. Anti-p200 pemphigoid, a rare subset AIBD, was first described by Japanese researchers in 1996. This disease is characterized by the presence of autoantibodies targeting a 200-kDa dermal protein located in the lower lamina lucida of the basement membrane zone. Notably, approximately 90% of sera from patients with anti-p200 pemphigoid exhibit immunoreactivity to the γ-1 chain of laminin. Consequently, some researchers have proposed renaming this condition to anti-laminin γ1 pemphigoid [30].

The mean age of patients with anti-laminin γ1 pemphigoid is 65.5 years, with a range spanning from 5 to 94 years, which is notably younger compared to individuals with BP. Patients with anti-lamγ1 pemphigoid typically present with bullae, urticarial plaques, and occasional pruritus, which can resemble other subepidermal autoimmune bullous diseases, particularly BP. Mucosal lesions have been reported in 20% to 40% of these patients [31].

It is known that anti-lamγ1 pemphigoid patients often have pre-existing psoriasis. A recent systematic review reported a prevalence of psoriasis in 56.0% of Japanese patients with anti-lamγ1 pemphigoid, compared to 6.4% in non-Japanese cases. Given this strong association between anti-lamγ1 pemphigoid and psoriasis, it is reasonable to consider the potential development of anti-lamγ1 pemphigoid in psoriasis patients who present with bullae. However, the mechanisms underlying the frequent coexistence of these two conditions and the specific clinical characteristics of these patients remain inadequately understood [31].

The association between pemphigus and psoriasis was only recently suggested by a few epidemiological studies. Three cross-sectional studies examined the prevalence of psoriasis among patients with pemphigus compared to control groups. A meta-analysis of these studies found a pooled odds ratio (OR) of 3.5 for psoriasis in patients with pemphigus.

Pemphigus refers to a group of rare, potentially serious autoimmune blistering disorders that affect the intraepidermal layer of the skin. Clinically, it presents with vesicles and erosions on the skin and mucous membranes, which can significantly impact the patient’s quality of life and lead to increased morbidity and mortality. The condition’s pathogenesis involves the production of immunoglobulin G (IgG) autoantibodies targeting desmoglein 3 and desmoglein 1, which are cadherin-type cell–cell adhesion molecules in the epidermis.

There are fewer reports of pemphigus foliaceus being associated with psoriasis. Studies indicate that patients with psoriasis are more than three times as likely to develop new-onset pemphigus foliaceus compared to the control group. Additionally, across all published studies, the incidence of comorbid psoriasis in patients with pemphigus foliaceus is 2.4%. The most commonly associated types of pemphigus foliaceus with psoriasis are pemphigus erythematosus, followed by pemphigus vulgaris, IgA pemphigus, and herpes-like pemphigus. The following hypotheses have been proposed to explain their co-occurrence, as seen in Table 3 [32].

Nevertheless, the pemphigus group is associated less frequently with psoriasis, compared to the autoimmune subepidermal bullous diseases group [31].

Connective tissue diseases, including systemic lupus erythematosus (SLE), systemic sclerosis (SSc), and dermatomyositis (DM), also display a complex relationship with psoriasis [32].

Both psoriasis and SLE belong to a group of immune-mediated inflammatory diseases (IMIDs), but their coexistence is very rare. Studies are limited to case series and small, single-center retrospective studies. The prevalence of SLE in patients with psoriasis has been reported to be estimated at 0.69%, while psoriasis occurs in 1.1% of patients who had previously been diagnosed with lupus, which is rarer than in the general population [33]. The types of psoriasis were plaque-type (87.3%), pustular type (4.8%), scalp psoriasis (7.9%), and psoriatic arthritis (PsA) (1.6%), suggesting that the co-existence of SLE and PsA is rare. Since SLE is more prevalent in women, the coexistence of psoriasis and SLE affects women more often as well, with a 1:5.8 ratio versus 1.3:1 in patients with psoriasis but without LE [34]. As compared with SLE, association of chronic cutaneous LE, such as LE profundus, has been rarely reported in association with psoriasis [35].

Several similarities have been observed between psoriasis and SLE, including genetic, epigenetic, and pathogenic factors. The shared pathomechanisms include innate immunity, type I interferon, plasmacytoid dendritic cells, neutrophil extracellular traps, and Th1/Th17-type cytokines [1]. IL-17, IL-22, and IL-23, produced by Th17 cells, have been shown to play a major role in maintaining chronic inflammation in both psoriasis and SLE, and an upregulated Th17 immunologic response may explain the association between both conditions, as well as provide a therapeutic target for management of concomitant psoriasis and SLE [36].

Genome-wide association studies have found that SLE and psoriasis share common genetic susceptibility loci, such as MHC genes, and non-MHC, such as PTPN22, STAT4, and TNIP1. Recent case–control differential analysis by national scholars has identified NFKBIA and IL28RA as common susceptibility genes for psoriasis and SLE. These findings support the potential co-occurrence of psoriasis and SLE [37]. Moreover, LL37, an endogenous antimicrobial peptide, has been implicated in both SLE and psoriasis. LL37 triggers IFN-α production in plasmacytoid dendritic cells, and SLE patients had circulating T-cells responding to LL37, which correlated with anti-LL37 antibodies and disease activity. Compared with psoriasis, LL37-specific T-cells in SLE displayed a T-follicular helper-like phenotype, implicating a pathogenic role in SLE [38].

Cases of SLE following PsA or vice versa are uncommon. Due to the complexity of autoimmune diseases and the rarity of such cases, comprehensive global data on the co-occurrence of these conditions is limited. The key interleukin IL-17, with IL-17A being particularly influential, appears to play a significant role in the progression of both diseases. Venetsanopoulou et al. reported seven cases of SLE and PsA coexistence. In five out of seven, PsA occurred before the development of SLE, while in the remaining two cases, SLE was diagnosed before PsA [39].

Managing patients with both SLE and psoriasis is complex. The medications must be meticulously assessed to prevent the worsening of either condition and to reduce associated morbidities. One of the main treatment methods for psoriasis, UV radiation, may induce and/or exacerbate not only skin lesions but also the course of SLE [40].

In turn, antimalarial drugs, which are fundamental in the treatment of SLE, may induce or exacerbate psoriatic skin lesions and have even been linked to the induction of psoriasis. Midorikawa et al. reported a case of annular pustular psoriasis induced by hydroxychloroquine in a patient with a gain-of-function mutation in caspase recruitment domain family member 14 (CARD14). Several mechanisms have been proposed, including the suppression of the epidermal barrier function via inhibition of transglutaminase activity, induction of keratinocyte proliferation via IL-17 production, and interference with cholesterol metabolism. The gain-of-function CARD14 mutation causes psoriatic disease through the activation of nuclear factor-κB, suggesting the involvement of CARD14 mutation in the pathogenesis of APP. In this case, hydroxychloroquine administration was considered to have induced APP based on CARD14 mutation, a genetic predisposition for psoriatic diseases, but the relation between SLE and CARD14 mutation remains to be elucidated [41].

Regarding biologics, the development of SLE as a result of treatment with TNF-α inhibitors is relatively rare—0.19–0.22% for infliximab, 0.18% for etanercept, and 0.10% for adalimumab—and the autoantibodies induced are typically IgM and are not pathogenic [42].

The biologics targeting the Th17 pathway, including ustekinumab and secukinumab, have been successfully used in the treatment of patients with concomitant psoriasis and LE. Dai et al. report the case of a 23-year-old female patient with psoriasis and SLE that improved after receiving secukinumab treatment [34]. However, several case reports presented new-onset SLE or the deterioration of pre-existing SLE during or after Th17-targeted treatment [43]. Hsieh et al. reported a patient with aggravation of discoid lupus erythematosus (DLE) after secukinumab treatment for psoriasis. Symptoms of psoriasis and PsA almost resolved after 150 mg secukinumab every four weeks for 2 years, but lesions of DLE enlarged and became generalized [44]. A few cases of concomitant psoriasis, PsA, and SLE have been successfully treated with ustekinumab, following advances in research into the roles of IL-17 and IL-23 in its pathophysiology [45]. Ustekinumab has shown a positive response in treating cutaneous lupus, including subacute cutaneous lupus erythematosus (SCLE) and DLE, regardless of the presence of concomitant psoriasis. A randomized, placebo-controlled, phase II trial involving 102 patients with active SLE reported that ustekinumab, when added to standard therapy, significantly improved the SLE Responder index (SRI-4), with 60% of patients achieving this outcome compared to 31% in the placebo group. Additionally, significant reductions in the SLEDAI-2K were observed at week 24. Ustekinumab shows promise as a treatment for SLE and could be a valuable option for patients with concurrent SLE and psoriasis. However, despite positive reports regarding the effectiveness of ustekinumab in treating cutaneous LE and SLE, there have been instances of adverse reactions associated with the treatment. For example, Goh et al. reported a case of a patient with both psoriasis and SLE who experienced exacerbations of cutaneous LE and developed acute class IV lupus nephritis after receiving five doses of ustekinumab [46].

Dermatomyositis (DM) is a rare inflammatory disease that presents distinct cutaneous manifestations and various degrees of potentially life-threatening systemic involvement, such as pulmonary diseases or malignancies [41].

Psoriasis and DM rarely coexist, with only a few cases documented. Chu et al. reported a case of psoriasis combined with DM, and a literature review was performed to speculate on the possible pathogenesis and treatment. In these cases, five were male and ten were female, of which six patients were under 30 years old, and they were all diagnosed with juvenile DM. In six cases, psoriasis occurred before DM, whereas in other cases, DM developed first. Among these cases, one had diabetes, hypertension, and antiglomerular basement membrane disease, another had a hepatic tumor, a third had Hashimoto’s thyroiditis and Sjögren syndrome, and a fourth had interstitial lung disease. Regarding the possible triggers, three cases might be associated with medications, including adalimumab, secukinumab, and withdrawal of prednisolone. One case might have been linked to a hepatic tumor. The patient had no history of medication use or underlying disease. He was a farmer who had been exposed to prolonged sunlight without any protection in the summer. The authors suspected that sunlight exposure could be a possible trigger [47].

Referring to the pathogenesis, sufficient evidence suggested that DM and psoriasis shared some common inflammatory pathways, including IFN-α/β-induced response, INF-γ, and TNF-α. In addition, new studies have proved that pDCs are located in the epidermis of DM, which is shared with the pathogenesis of psoriasis. Expression of IL-17 in the cellular infiltrates in both skin lesions may suggest that IL-17 possibly contributes to the development of DM as well as psoriasis [48]. While psoriasis and DM share certain signaling pathways and cytokines, the exact mechanisms behind their co-existence are still not well understood. Based on previous studies, there seems to be a complex, interactive, and self-sustaining inflammatory circuit among these cytokines in psoriasis. As mentioned in the case above, sunlight exposure might play a significant role in the pathogenesis of the co-existence of DM and psoriasis. UV radiation could suppress the IL-23/IL-17 axis, resulting in the inhibition of the production of IL-17. This immune response results in a reduction of IL-17-mediated inflammation in skin lesions, which is similar to the effect of IL-17 inhibitors, and can also disrupt the inflammatory circuit. So, while ultraviolet phototherapy is safe and effective for psoriasis, it is considered a trigger for DM and even exacerbates the symptoms of DM. On the other hand, systemic corticosteroids, which are considered first-line therapy for the management of DM, are not recommended for use in cases where psoriasis coexists with DM unless the situation is highly critical and the symptoms cannot be controlled by other therapies, due to concern regarding the potential exacerbation of psoriasis after corticosteroid withdrawal [33].

Adalimumab, a TNF-α inhibitor, secukinumab, an IL-17 inhibitor, and ustekinumab, which targets interleukin IL-12/23p40, are effective treatments for psoriasis and have shown potential in treating psoriasis concomitant with DM. Nevertheless, secukinumab has been reported as a triggering factor for psoriasis concurrent with DM. This has led to caution in using biologics targeting the IL23-IL17 pathway to treat this condition. The medication targeting the type II IFN-mediated signaling pathway could be a therapeutic alternative for psoriasis concurrent with DM. The type II IFN-mediated signaling pathway is regulated by JAK 1 and JAK2, and there are several JAK inhibitors targeting this pathway, including tofacitinib, ruxolitinib, baricitinib, abrocitinib [48].

Recently, Xu et al. reported a case of refractory psoriasis with DM demonstrating a positive response to oral upadacitinib, and the treatment was well tolerated. Since the JAK1-STAT pathway plays a critical role in psoriasis and DM, upadacitinib, a highly selective JAK1 inhibitor, may be an effective and safe option for treating refractory concomitant skin autoimmune diseases with JAK1- STAT pathway activation [48].

Due to the complexity of autoimmune diseases and the rarity of such cases, further studies are required to find the optimal treatment in patients suffering from both DM and psoriasis.

Systemic sclerosis (SSc) is an uncommon autoimmune condition marked by a characteristic triad: vasculopathy, skin and internal organs fibrosis, and immunologic dysfunction. Although clinical signs and symptoms such as Raynaud’s phenomenon are prototypical of SSc, this disease globally exhibits a wide range of clinical manifestations with a high inter-individual variability [49].

Localized scleroderma, also known as morphea, is an autoimmune disease characterized by fibrosis, primarily affecting the skin and subcutaneous tissue, with occasional involvement of the underlying bone.

The association between psoriasis and morphea is currently unknown, and the co-occurrence of these conditions is believed to be quite uncommon, with only 22 cases documented in the literature. Psoriasis has been confirmed in 11.6% of documented immune-mediated conditions seen in conjunction with morphea, making it one of the most common co-occurring autoimmune conditions [50].

Cases of co-existing SSc and psoriasis have been also reported, suggesting a possible association between the two conditions. The possible link between SSc and psoriasis has been further evaluated in a cohort study from Israel. In that research, 2431 patients with SSc were evaluated with 12,710 age- and sex-matched controls. Psoriasis was more common in patients with SSc (1.9%) than in controls without SSc (1.2%) [51].

Several hypotheses or mechanisms have been proposed to explain the potential link between SSc and psoriasis. Studies have shown associations between MHC-I molecules—particularly HLA-B*08:01—in both SSc and psoriasis, specifically psoriatic arthritis, due to shared genetic background. Furthermore, both diseases share certain immunopathogenic aspects. In psoriasis, skin inflammation is dominated by CD4+ and CD8+ T-cells, with CD8+ T-cells emerging as key players. In SSc, CD4+ T-cells, including follicular helper T-cells, may drive autoantibody production and interactions between CD4+ Th2 T-cells and fibroblasts releasing profibrotic mediators (IL-4, IL-6, IL-13), which may contribute to the skin phenotype [50].

The development of both conditions has been linked to type I interferon pathways, as described in studies involving patients who received intense interferon therapy for hepatitis C infection, multiple sclerosis, and myeloproliferative diseases.

Another significant mechanism connecting SSc and psoriasis is the skin target tissue damage, such as radiation and direct trauma, which exposes neo-autoantigens to the immune system and may initiate local adaptive immune reactions [52].

Cases of biologic treatment-induced morphea are a rare complication, with only 10 cases reported in the literature, 2 of which involved patients treated with ustekinumab. Shaffer et al. presented a patient who received ustekinumab treatment for psoriasis with resolution but exhibited subsequent development of sclerotic nodules consistent with morphea and whose lesions resolved once the medication was stopped. The disease mechanism is not fully known but is likely related to the profibrotic activity of Th2 cells [52].

Cirone et al. described a patient with long-standing plaque psoriasis under ustekinumab who developed morphea under a plaque of psoriasis [53].

Both morphea and psoriasis have a common immunologic basis involving inappropriate immune activation against self-antigens, which provides a possible explanation for their coexistence. The Th17 pathway and cytokines IL-17, IL-21, and IL-23 are believed to significantly impact the pathogenesis of inflammatory autoimmune conditions. The Th2 pathway in morphea and the Th1 pathway in psoriasis, both intersect with the Th17 pathway. Thus, it is postulated that dysregulation of this pathway may lead to the coexistence of these conditions in the same patient [51].

Elias et al. reported the case of a patient who developed biopsy-proven scleroderma after initiation of secukinumab for her psoriatic arthritis. The administration of secukinumab is temporally associated with skin changes without the influence of any confounding medications. The duration between drug exposure and the onset of skin lesions was 19 months [51].

To understand the cause of their dual presentation, it is important to have additional reports of similar cases that demonstrate the coexistence of psoriasis and morphea in patients treated or not with biologics, as well as an improved understanding of their pathophysiology.

**Figure 1:**
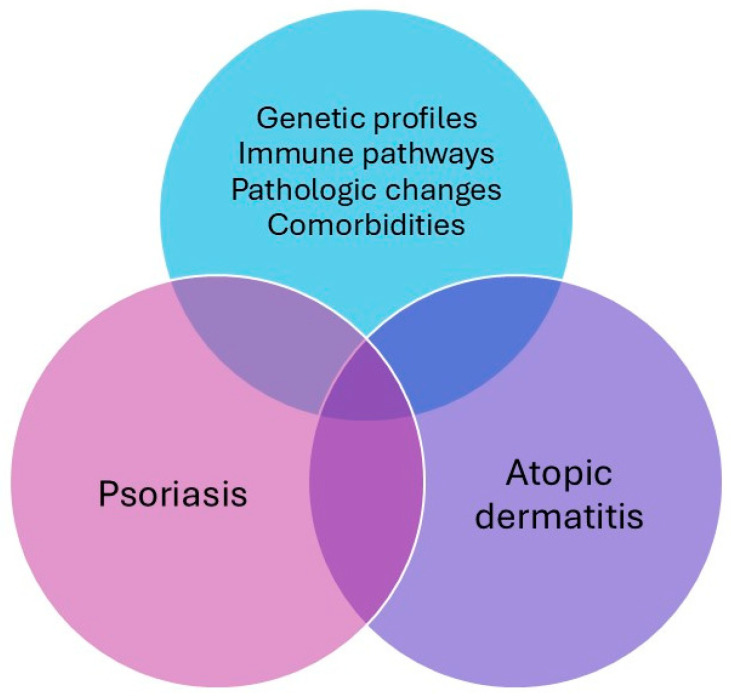
Shared features between psoriasis and atopic dermatitis.

Psoriasis and atopic dermatitis (AD) are common inflammatory skin conditions with distinct clinical features and immunological profiles. Overlapping conditions could be classified into mainly psoriasis lesions with AD features or vice versa, concomitant psoriasis and AD, or disease transformation as a result of biologics treatment. Psoriasis typically affects the scalp and extensor surfaces, while AD varies with age: it affects the extensor surfaces of extremities and the face in infancy, and the flexural surfaces and hands in adolescents and adults. In terms of disease progression, psoriasis often begins around ages 20 to 30, with many patients experiencing lifelong symptoms. In contrast, AD typically starts in early childhood, often improving before adolescence (approximately 80% of the cases). Interestingly, studies show that individuals with psoriasis have a 25-fold lower prevalence of AD. There are shared genetic profiles, immune pathways, pathologic changes, and comorbidities for these two dermatological conditions (Figure 1). Additionally, it seems that individuals with abnormalities in their skin barriers and immune responses may be more prone to exhibiting the overlapping symptoms of psoriasis and AD [54].

In AD, exposure to various allergens triggers a systemic Th2 immune inflammation termed epithelial susceptibility. Furthermore, the interaction between Th2/Th17-related cytokines and skin structures or Kupffer cell cytokines determines a cycle or loop of skin barrier–inflammatory cytokine interactions. The overlap between AD and psoriasis starts with a Th2 inflammatory activation following the breakdown of the skin barrier due to genetic or other factors. In psoriasis, the skin barrier function may be compromised due to trauma (intense scratching), a genetic mutation affecting epidermal barrier integrity, and the downregulation of barrier-related proteins because of dysregulated Th17-related cytokines [55].

Both psoriasis and AD show significant genetic influences, evidenced by familial patterns and greater disease risk in identical twins compared to fraternal twins. Numerous genetic loci have been identified for each condition, highlighting their genetic diversity. The most common susceptibility gene for psoriasis is HLA-Cw*0602, located on PSORS1 at 6p21, while null mutations in the filaggrin (FLG) gene represent the strongest genetic risk factor for developing atopic dermatitis. The epidermal differentiation complex on chromosome 1q21.3 contains FLG gene mutations for AD, and it seems to increase the risk of developing psoriasis in Taiwanese and Chinese populations [56,57]. Another common genomic region is located on chromosome 5q31.1-q33.1, where IL-13 has been linked to both AD and psoriasis [58].

Psoriasis primarily involves the TNF-α and the IL23-Th17-IL17 pathway, while AD is associated with a Th2 response driven by IL-4 and IL-13. However, the Asian, pediatric, and intrinsic forms of AD also show Th17 involvement. Research on psoriasis susceptibility genes found that there is a 1.18-fold increase in loci related to IL-4/IL-13 signaling. Additionally, both conditions involve Th1 and Th22 responses. Interestingly, despite elevated IL-22 levels in both diseases, it seems that blocking IL-22 lacks efficacy in psoriasis and has modest results in AD treatment [59].

Overlapping manifestations and phenotypes between psoriasis and AD have been increasingly reported. Immunopathogenesis, common to both conditions, is supported by clinical features.

IgE is commonly used as a marker for AD. However, total IgE levels are notably higher in psoriasis patients compared to healthy individuals. Moreover, those with long-standing psoriasis also show a significant increase in total IgE levels.

In vitro studies show that scratch injuries in both conditions lead to CCL20 production by keratinocytes, which, in turn, attracts IL17-producing immune cells, contributing to the elevated IL-17 levels observed in AD as well. However, the mechanisms behind itch differ between the two diseases. In psoriasis, pruritus involves Substance P, IL-2, calcitonin gene-related peptide (CGRP), μ-opioid receptors (OPRM), and κ-opioid receptors (OPRK), while in AD pruritus is linked to thymic stromal lymphopoietin (TSLP), CGRP, IL-4, IL-13, and IL-31. In psoriasis, pruritus is primarily driven by the transient receptor potential vanilloid 1 (TRPV1) channel, whereas AD itch is largely mediated by the transient receptor potential ankyrin 1 (TRPA1) channel [60].

There is evidence that prostanoids and leukotrienes (LTs) are produced at significantly higher levels in the lesional skin of chronic inflammatory conditions like AD and psoriasis. These lipid mediators are generated when arachidonic acid, released from membrane phospholipids in response to various pathophysiological stimuli such as trauma, is converted into prostanoids and LTs. Specifically, compounds like PGE2, LTB4, and CysLTE4 have been detected in the lesional skin or urine of patients with AD, suggesting their involvement in the disease’s progression.

The pathological processes underlying AD can be categorized into three main components: (i) disruption of type 2 immunity; (ii) impairment of the skin barrier; and (iii) presence of itch. In AD, there is a notable elevation of type 2 cytokines like IL-4 and IL-13, which contribute to various clinical manifestations such as barrier dysfunction and itch. For instance, IL-4 and IL-13 are known to impede the production of filaggrin, a crucial protein for maintaining skin barrier function, in keratinocytes. Additionally, signaling from IL-4 and IL-13 receptors in neurons is crucial for sustaining chronic itch. In AD lesions, IL-4 and IL-13 are mainly secreted by Th2 cells, although other innate cells like group 2 innate lymphoid cells (ILC2), basophils, and mast cells also contribute to type 2 cytokine production in the skin. Studies have shown the involvement of prostanoids in the induction of Th2 cells in AD models. For instance, the absence of COX-2 or inhibition of its activity led to enhanced eosinophil infiltration and increased IL-4 levels, indicating a regulatory role of prostanoids in type 2 immune responses [61].

Epicutaneous sensitization with protein antigens highlights the significant role of Langerhans cells (LCs) in inducing allergic responses. The modulation of prostaglandin (PG) receptors during sensitization influences the development of AD-like skin lesions, suggesting the regulatory role of PG signaling in AD pathogenesis. Various prostanoids and receptors are implicated in the fine-tuning of T-cell differentiation in AD. IL-22 is suspected to contribute to epidermal hyperplasia in AD lesions, and PGE2-EP4 signaling has been shown to regulate IL-22 production. Moreover, CCL17/TARC production, a key Th2 chemokine in AD, is promoted by PGE2, showcasing the diverse functions of prostanoids in AD pathogenesis. Prostanoids like PGD2 play a role in recruiting type 2 immune cells to AD skin lesions through CRTH2 signaling. LTs, particularly LTB4 acting on BLT1 receptors, are involved in Th2 cell infiltration in AD lesions. PG, especially PGD2, potentially plays a role in the recruitment of type 2 immune cells in the skin lesions of AD. Activated MCs are the primary source of PDG2 production. The receptor CRTH2, crucial for PGD2, is expressed on various type 2 immune cells like Th2 cells, eosinophils, and basophils. CRTH2 stimulates chemotaxis and degranulation in Th2 cells, eosinophils, and basophils. Recent findings suggest that human ILC2 cells also express CRTH2, and the PGD2-CRTH2 signaling pathway facilitates cytokine production and chemotaxis in ILC2 cells. In AD patients, an increase in circulating CRTH2+ cells has been observed [62].

LTs may also contribute to Th2 cell infiltration in AD lesions through LTB4-BLT1 signaling. BLT1, the receptor for LTB4, is expressed in various cells, including neutrophils and Th2 cells. Scratching leads to neutrophil infiltration in the skin, where LTB4 produced by these neutrophils acts on BLT1 receptors expressed on neutrophils and Th2 cells, aiding their infiltration into the skin. Studies have shown the therapeutic potential of 5-lipoxygenase inhibitors in managing AD, indicating the involvement of LTB4 in AD progression. Additionally, cysteinyl leukotrienes (CysLTs), particularly CysLTE4, produced abundantly by skin mast cells, promote Th2 cytokine production via the CysLT1 receptor. These CysLTs also induce migration, cytokine production, and receptor expression in ILC2 cells. The effects of CysLTE4 on Th2 and ILC2 cells are synergistically boosted by PGD2. Therefore, targeting PGD2 and CysLTE4 or their receptors collectively may present a novel therapeutic avenue for addressing AD.

Interestingly, PGE2 and PGD2 have been found to hasten the recovery of skin barrier function following mechanical scratching, suggesting a dual role for PGE2 mediated through different receptors in skin barrier maintenance. PGE_2_ facilitates pruritus in AD by inducing the release of histamine from mast cells.

Numerous prostanoids and LTs have been found in abundance in human psoriatic skin lesions. While their specific roles in psoriasis are not entirely understood, the use of advanced mouse psoriasis models, like the imiquimod-induced psoriasis model, has shed light on the involvement of these lipid mediators in psoriatic dermatitis. In this model, mice lacking TP receptors and wild-type mice treated with a TXA2 synthase inhibitor showed reduced dermatitis, along with decreased IL-17 production from γδ T cells in the skin. This suggests that TXA2-TP signaling may exacerbate psoriatic dermatitis by promoting IL-17 production in psoriatic skin lesions. Additionally, PGE2, another prostanoid, is implicated in promoting psoriatic dermatitis through the regulation of IL-23/IL-17 pathways [62].

IL-23 plays a crucial role in psoriasis development by stimulating the formation and activation of Th17 cells. Fibroblast-produced PGE2 has been shown to enhance IL-23 production from dendritic cells in vitro, supporting Th17 cell expansion. Moreover, PGE2 from Th17 cells boosts the expression of an IL-23 receptor subunit gene through EP2 and EP4, promoting Th17 cell generation. In a mouse psoriasis model induced by IL-23, deleting both EP2 and EP4 on T cells inhibited Th17 cell accumulation in the skin and halted psoriatic lesions development. Therefore, targeting PGE2-EP2/EP4 signaling may hold promise as a therapeutic strategy for reducing Th17 cells in psoriasis. The potential roles of LTs in psoriasis have also been explored. In the imiquimod-induced psoriasis model, LTB4-BLT1 signaling in neutrophils expedited their skin infiltration by cooperating with CXCR2 and intensified psoriasis development. Additionally, LTB4 influenced skin DCs and γδ T cells through BLT1, enhancing their migration and cytokine production, and contributing to psoriatic dermatitis progression. The LTB4-BLT1 pathway may play a part in the pathogenesis of psoriasis through these mechanisms [62].

It has been proven that both psoriasis and AD are closely related to obesity. The broad range of cytokines and chemokines, along with adipokines, leptin, and fatty acids, leads to the exacerbation of psoriasis and AD and a significant reduction in treatment effectiveness. The stress experienced during adipose tissue expansion in obesity may stretch the epidermis, resulting in a compromised epidermal barrier function and triggering the innate immune response, which can initiate autoimmune activation. Proinflammatory molecules, including free fatty acids, adipokines, and cytokines released from expanding adipose tissue, can activate innate immune cells such as neutrophils, macrophages, and dendritic cells. This activation leads to the stimulation of Th1, Th17, and/or Th22 cells, ultimately resulting in epidermal hyperplasia. Adiponectin, an anti-inflammatory adipokine, normally inhibits the activation of myeloid and lymphocyte cells. However, in obese individuals, the expression of ADIPOQ is diminished in hypertrophied adipocytes, which may play a role in the development of psoriasis and/or atopic dermatitis. A compromised skin barrier can allow allergens and pathogens to enter, fostering a Th2 immune response that drives acute allergic inflammation and contributes to the pathogenesis of atopic dermatitis [62].

Psoriasis is classified as a T cell-mediated disorder, as T cell-derived cytokines like IL-17A and IL-22 are responsible for the hyperproliferation and abnormal differentiation of keratinocytes, leading to the formation of psoriatic plaques. However, the recognition of DAMPs or PAMPs by pattern recognition receptors (PRRs) in keratinocytes or plasmacytoid dendritic cells (pDCs) is believed to trigger the initial events in psoriasis. These events set off innate immune responses that drive the later development of adaptive immunity and autoimmunity. In response to DAMP or PAMP stimulation, keratinocytes can produce a range of proinflammatory cytokines, including IFN-β, IL-1β, IL-36, TNF, IL-6, IL-8, IL-25, and CXCL10, which help establish the inflammatory T cell phenotype characteristic of psoriasis [63].

PRR-mediated activation of innate immune responses in keratinocytes contributes to the onset of psoriasis following skin injury. When the skin is damaged, cells release DAMPs such as dsRNA, ssRNA, and DNA. Notably, the antimicrobial peptide LL37, which is upregulated during wounds, facilitates the recognition of dsRNA in keratinocytes via the TLR3 and mitochondrial MAVS (mitochondrial antiviral signaling protein) pathways, leading to IFN-β production by keratinocytes or pDCs. It was demonstrated that LL37 enables pDCs to recognize ssRNA or DNA through TLR7 or TLR9, resulting in significant IFN-α production. Furthermore, self-ssRNA-LL37 complexes activate conventional dendritic cells via TLR8, promoting the release of TNF-α and IL-6 and aiding in cDC maturation.

The roles of TLR2 and TLR4 in psoriasis remain ambiguous. TLR2 and TLR4 expressions are heightened in peripheral blood mononuclear cells and keratinocytes of psoriasis patients. Additionally, polymorphisms within TLR4 have been linked to chronic plaque psoriasis and psoriatic arthritis. Recent studies indicate that neutrophil infiltration into the epidermis triggers inflammatory responses by activating the epidermal TLR4-IL36R crosstalk in an imiquimod-induced psoriasis-like mouse model. Moreover, heat shock proteins (HSPs) such as HSP27, HSP60, HSP70, and HSP90 are overexpressed in psoriasis keratinocytes, acting as autoantigens that activate antigen-presenting cells via TLR4, promoting their maturation and the secretion of TNF-α and IL-12 [63,64].

The skin barrier-inflammatory pathway plays a role in driving the transition from psoriasis to atopic dermatitis. The skin barrier is formed by physical components such as keratinocytes, keratin, cornified cell envelope (CE), intercellular lipids, and skin connective structures, as well as chemical components such as antimicrobial peptides and natural moisturizing factors (NMFs). Any trigger that affects these structures can predispose to a Th2 immune response.

In the psoriasis–AD overlap, cytokines linked to Th1, Th17, and Th22 responses in psoriasis help downregulate the expression of filaggrin (FLG), loricrin (LOR), and involucrin (IVL). This reduction in FLG activates the Th2 immune axis, intensifying the inflammatory response. Moreover, the Th2 immune axis can also decrease the expression of FLG, LOR, and IVL, creating a feedback loop between the epidermal barrier and inflammatory factors. This dysregulation of both the epidermal barrier and immune response worsens the disease and extends the chronic nature of AD. This reciprocal interaction between T cells and the cornified envelope may be a crucial focal point for understanding the interplay between psoriasis and AD and the chronicity of both conditions. Cytokines in psoriasis associated with AD seem to downregulate the expression of claudins (CLDNs), disrupting the skin barrier. Furthermore, this impairment can accelerate Th2-type inflammation. CLDNs are integral to both Th2 and IL-1β inflammatory pathways in the context of psoriasis–AD overlap [64,65,66].

## 5. Conclusions

The development of psoriasis is multifactorial, resulting from a combination of genetic predisposition, environmental triggers, and autoimmune pathogenic traits. Similarly, skin conditions related to psoriasis also display a labyrinthine pathophysiology, in which genetic, immune, and environmental factors are markedly involved.

In recent years, significant advances have been made regarding the complex pathophysiology of psoriasis, resulting in a better understanding of the disease and its multifaceted connections with other cutaneous diseases such as AA, HS, vitiligo, bullous dermatoses, autoimmune diseases, and AD.

While distinct in their clinical presentation, the skin diseases related to psoriasis may become associated, complicating diagnosis and treatment. Understanding the intricate interconnection between psoriasis and these conditions is of interest to scientists in developing novel research directions and to clinicians in providing holistic care, as managing one condition may influence the course of others.

Among the future therapeutic directions are biologic therapies, including TNF-α inhibitors, as well as anti-IL-17 and anti-IL-12/23 agents, which show promising results in psoriasis and overlapping conditions. Similarly, the combined use of biologic therapies has been proposed as a potential strategy for treating associated conditions, such as psoriasis with AA, vitiligo, AD, or bullous diseases. While this approach targets multiple disease mechanisms and could improve symptom control and overall management of complex conditions, further research is needed to refine these combinations and ensure their safety and efficacy.

Another possible direction could involve dysfunctional Tregs, which are implicated in both psoriasis and related autoimmune diseases. Tregs are crucial for maintaining immune system balance, and their dysfunction may contribute to the development of these conditions. Therapies aimed at enhancing the function of Tregs could be beneficial across multiple autoimmune diseases, as they may help restore immune regulation and prevent the excessive inflammatory responses that characterize conditions like psoriasis, as well as other related diseases.

Granzyme B inhibition and autophagy restoration represent promising therapeutic approaches for managing psoriasis and AA. Granzyme B plays a role in inflammation and tissue damage in these conditions, so targeting this protease could help mitigate their effects. Additionally, enhancing autophagy may preserve cellular health and control inflammation, further improving disease management.

Other therapeutic approaches include targeting the JAK-STAT signaling pathways, which are critical in conditions like AD, DM, and psoriasis, through selective JAK inhibitors such as upadacitinib.

Mast cell stabilizers, such as cromolyn sodium and related treatments, show potential in managing drug-induced urticaria and mast cell-associated inflammation in psoriasis, offering a therapeutic approach to address these specific aspects of the condition.

Therapies focused on skin barrier restoration and microbiome modulation offer a promising approach for addressing the overlap between psoriasis and AD, as well as conditions like HS. Improving the skin barrier and targeting disruptions in the skin microbiome could help restore balance, reduce inflammation, and enhance disease management across these related conditions.

Growing interest surrounds alternative treatments for managing psoriasis and other chronic skin conditions, especially those leveraging natural bioactive compounds. Substances like flavonoids, terpenoids, omega-3 fatty acids, and alkaloids demonstrate significant potential due to their ability to modulate immune responses, reduce inflammation, and combat oxidative stress. Emerging research highlights their effectiveness in easing symptoms like inflammation and scaling, both as independent treatments and as adjuncts to traditional methods. Despite their promise, further investigation through rigorous clinical trials is essential to standardize their use, ensure long-term safety, and maximize their therapeutic potential.

## Figures and Tables

**Table 1:** Risk factors associated with both the onset and exacerbation of psoriasis.

Intrinsic Factors	Extrinsic Factors
Metabolic syndrome	Mechanical trauma (Kӧbner phenomenon)
Emotional stress	Alcohol
Dyslipidemia	Smoking
Hypertension	Infection
Diabetes	Vaccination
Obesity	Drugs
	Organochlorine and organophosphate compounds

**Table 2:** Systemic medication associated with the onset and exacerbation of psoriasis.

Medications Involved in Triggering and/or Exacerbating Psoriasis
Beta-blockers
Angiotensin-converting enzyme inhibitors
Nonsteroidal anti-inflammatory drugs
Antimalarials
Lithium
Interferons
Tetracycline
Terbinafine
Imiquimod
Fibrate drugs
Biologic therapy

**Table 3:** Hypotheses explaining the co-occurrence of psoriasis and pemphigus foliaceus.

Hypothesis	Observations
Plasminogen Activation Hypothesis	The activation of the plasminogen system has been suggested as a factor in the acantholysis observed in pemphigus. Elevated levels of tissue plasminogen activator have been noted in psoriatic skin lesions, potentially linking the two conditions.
Common HLA Antigen	The human leukocyte antigen (HLA) DRB1 alleles are involved in both psoriasis and pemphigus foliaceus. A shared HLA genotype may lead to abnormal T lymphocyte activation, resulting in the manifestation of lesions characteristic of both diseases.
Decreased Suppressor T Lymphocyte Function	This reduction of suppressor T-lymphocyte function can lead to increased activity of the humoral immune system and the production of autoantibodies, contributing to the pathogenesis of both conditions.
Auto-Inflammation and Autoimmunity Link	The pathogenesis of psoriasis involves both auto-inflammation and autoimmunity. It has been established that auto-inflammatory diseases can be linked with autoimmune disorders. Elevated levels of IL-1β, common in auto-inflammatory conditions, may activate adaptive immunity and thus trigger autoimmune responses.
Role of NLRP1 Inflammasome	Variations in the NLRP1 gene, which encodes proteins involved in the inflammasome, have been associated with increased susceptibility to both psoriasis and pemphigus foliaceus. This genetic variation could represent a common pathogenic mechanism underlying both diseases.
